# Avian Infectious Bronchitis Virus: Molecular Detection in Southwestern Ethiopia Chickens

**DOI:** 10.1155/2024/6979448

**Published:** 2024-09-14

**Authors:** Bezina Arega Emeru, Haregawi Tesfaye Desta

**Affiliations:** ^1^ National Agricultural Biotechnology Research Center Ethiopian Institute of Agricultural Research, Holeta, Ethiopia; ^2^ College of Agricultural and Veterinary Medicine Jimma University, Jimma, Ethiopia

## Abstract

Infectious bronchitis virus (IBV) is a significant threat to poultry worldwide, but its status in Ethiopia remains understudied. Thus, this study aimed to detect the virus and associated risk factors in South West Ethiopia. Ninety oropharyngeal swab samples were purposively collected from symptomatic chickens located in Jimma town, Seqa Chekorsa, and Tiro Afeta woredas of the Jimma zone between November 2021 and April 2022 to detect IBV virus by using RT-PCR. A side-by-side questionnaire was administered to assess risk factors. Total RNA was extracted, reverse transcription polymerase chain reaction (RT-PCR) was conducted, and products were visualized under UV light. The overall proportion of IBV was 16.6% (15/90). No statistical association was observed between any of the animal risk factors and the detection of the virus (*P*=0.57, 0.586, and 1). However, the proportion of birds infected by the virus was higher in males, exotic breeds, and adults compared to females, local breeds, and young birds. Similarly, none of the management risk factors had a significantly different effect on virus detection (*P*=0.25, 0.09, 0.088, and 0.726). However, improper carcass disposal (OR = 0.43, 95% CI: 0.13–1.4), lack of veterinary services (OR = 2.7, 95% CI: 0.8–8.3), and the presence of wild birds/rodents (OR = 4.4, 95% CI: 0.88-22.3) were associated with increased IBV risk but not cleaning of feeders/drinkers (OR = 1.1, 95% CI: 0.2–4.8). These findings underscore the need for enhanced biosecurity practices and further research to implement informed IBV control strategies in Ethiopia.

## 1. Introduction

Infectious bronchitis virus (IBV), classified within the genus *Gammacoronavirus* of the Coronaviridae family, is an archetypal avian coronavirus that infects domestic chickens (*Gallus gallus*). It causes infectious bronchitis, an acute, highly contagious respiratory disease in poultry, characterized by depression, snicking, coughing, head-shaking, and nasal and ocular discharges. Although primarily a respiratory disease, IBV also affects the female reproductive organs, leading to weak, cracked, or misshapen-shelled eggs with watery albumen. Some strains target the kidneys, causing nephritis and significant mortality [1].

Infections can be diagnosed by detecting IBV or specific antibody responses [2]. Reverse-transcription polymerase chain reaction (RT-PCR) targeting the 3′ untranslated region (UTR), N gene, and S1 and S2 parts of the IBV genome is commonly used for viral detection [3].

Since its identification as the causative agent of infectious bronchitis in 1936, IBV has spread globally [4]. It has been reported in North America [5], Europe [6], Latin America [7], Asia [8], and Australia [9]. In Africa, following its detection in Egypt in the 1950s [10] and Morocco [11], IBV has circulated widely, including in Nigeria, Niger [12], Burkina Faso [13], South Africa [14], Zimbabwe [15], and Kenya [16].

Since 2017, IBV has been reported in Ethiopia, detected through molecular methods [17, 18] and serology [19–21]. Despite its widespread presence and early discovery, IBV remains understudied, particularly in Ethiopia. This study aimed to determine the occurrence of infectious bronchitis virus (IBV) in diseased poultry and identify associated risk factors in southwestern Ethiopia using RT-PCR.

## 2. Materials and Methods

### 2.1. Study Area

**Figure 1:**
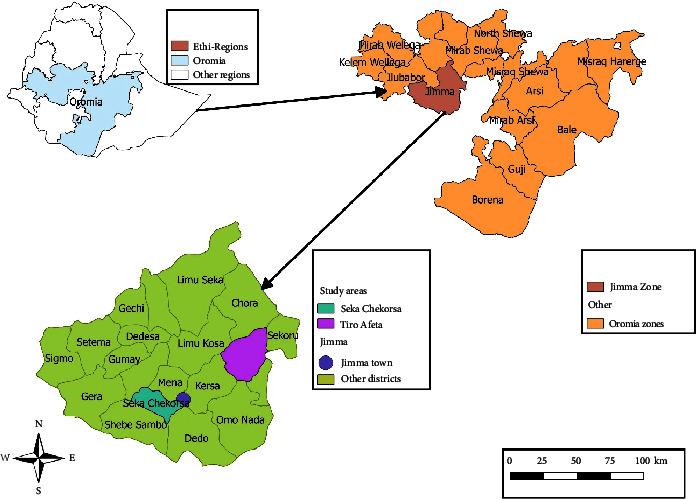
Map showing the study area.

Samples were collected from Jimma town, Seqa Chekorsa, and Tiro Afeta woredas of the Jimma zone, located 20 and 64 km from Jimma town, respectively (Figure 1). Jimma Town has an altitude of 1780 meters above sea level. Seqa Chekorsa and Tiro Afeta have altitude ranges of 2070–2220 and 1640–2800 meters above sea level, respectively [22].

### 2.2. Sample Collection, Study Design, and Risk Assessment

Ninety swab samples were purposively collected from symptomatic, IBV-unvaccinated, backyard, and small-scale chickens between November 2021 and April 2022. The sampled birds were brought to veterinary clinics in the study sites due to respiratory distress, ruffled feathers, or oral/ocular discharge. Oropharyngeal swabs were collected by inserting sterile, cotton-tipped swabs (Miraclean Technologies, China) into the oropharynx of the birds and gently rotating them against the walls. The swabs were stored in labeled, sterile universal bottles containing virus transport media (VTM). All collected swabs were transported to the National Agricultural Biotechnology Research Center Animal Biotechnology Research facility, Holeta, via cold chain, and preserved at −80°C until further processing. A semistructured questionnaire was administered to bird owners to assess associated animal and management risk factors (Appendix 1). Questions focused on flock and disease management practices. Outcomes were recorded and correlated with the virus occurrence rate. A total of 58 farmers were interviewed.

### 2.3. RNA Extraction and Reverse Transcription Polymerase Chain Reaction

The preserved samples were prepared for extraction after removal of gross contaminants and debris. Cryovials containing swab samples were thawed and centrifuged at 10,000 × g for 5 minutes. The supernatant was collected in a separately labeled 1.5 ml Eppendorf tube. Total RNA was extracted from 100 *μ*l of each sample using the DaAn Gene RNA Purification Kit (DaAn Gene Co., Ltd., China), according to the manufacturer's instructions. The extracted RNA was stored at −80°C for further analysis.

Nucleotide sequences for a set of primers with an amplicon product size of 149 bp were obtained from Rashid et al. [23], and a pair of primers was synthesized accordingly (Sigma-Aldrich, USA). The forward primer was 5′-GCT TTT GAG CCT AGC GTT-3′ and the reverse primer was 5′-GCC ATG TTG TCA CTG TCT ATT-3′. The annealing temperature and cycling conditions for the primers were optimized using the VaxSafe® IB Ingham Strain as the positive control.

Finally, reverse transcription and the polymerase chain reaction were conducted in a single reaction tube using the AccuPower Dual-HotStart RT-PCR Kit (Bioneer, Korea). Briefly, 1 *µ*l of each of the forward and reverse primers, 2 *µ*l of the extracted RNA as a template, and 16 *µ*l of nuclease-free water which added up to 20 *µ*l were dispensed into the master mix pellet and vortexed to create a homogenous mixture. Then, the mixture was placed in a PCR machine (Mastercycler® PCR thermal cycler, Germany), and the machine was set for thermal conditions of 51°C for 1 h for reverse transcription, followed by 95°C for 3 min, initial denaturation, and 36 cycles of 94°C for 20 s, 54°C for 20 s, and 72°C for 20 s, with a final extension step at 72°C for 5 min.

Finally, the products were visualized on a 1% agarose gel stained with ethidium bromide using a gel documentation system (BioDoc-It, USA) to detect the presence of the expected amplicon product in comparison with the reference 100 bp ladder (Bio Basic Inc., Canada).

### 2.4. Statistical Analysis

The data collected from the molecular tests were dichotomous. The responses recorded from the interviews were organized and trimmed for extreme values or homogenous responses. Finally, all adjusted data were analyzed for descriptive statistics, cross-tabulation (chi-square), and likelihood ratio analysis using SPSS V.26.

## 3. Result

**Figure 2:**
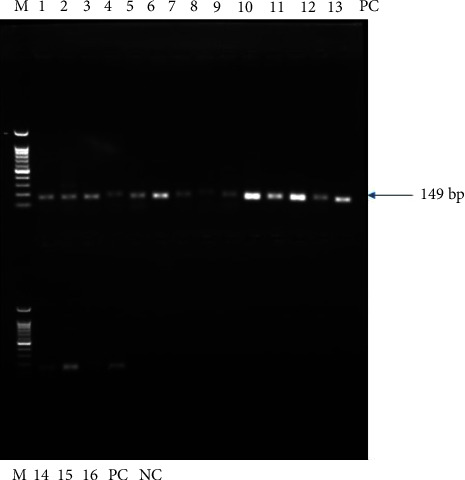
Detection of IBV using RT-PCR. Lane M: 100 bp molecular markers, lane 1–15 positive samples, 16 doubtful, PC: positive controls, and NC: negative control.

In this study, the overall proportion of IBV-positive samples detected using RT-PCR was 16.6% (15/90) (Figure 2). No statistical association was observed between any animal risk factors and virus detection (*P*=0.57, 0.586, and 1; 95% CI) (Table 1).

Bird sex was not associated with virus susceptibility. The proportion of infected birds and the likelihood of exposure were similar between males and females (14.3% vs. 19.5%; OR = 0.7; 95% CI: 0.2–2.0). Similarly, the distribution of the virus between exotic and local birds did not differ significantly (19% vs. 14.4%; OR = 1.3; 95% CI: 0.4–4.1). Age-based variation also showed no significant difference (15.9% for young birds and 17.4% for adults; OR = 0.9; 95% CI: 0.29–2.72).

Among the study sites, Seqa Chekorsa had the highest proportion of positive birds (20%), while Tiro Afeta had the lowest (11.5%).

None of the management risk factors significantly affected virus detection (*P*=0.25, 0.09, 0.088, and 0.726). However, birds from households that disposed of carcasses in open fields were twice as likely to be infected (22.2%) compared to those where carcasses were fed to pets (11.1%). The odds of infection were almost double in the first group (OR = 0.4; 95% CI: 0.13–1.4). Birds without treatment for various conditions were more than twice as likely to be infected (OR = 2.7; 95% CI: 0.8–8.3) compared to those receiving treatment. The presence of wild birds and rodents increased the risk of infection by more than fourfold (OR = 4.4; 95% CI: 0.88-22.3). However, cleaning poultry feeders or drinkers did not significantly affect virus distribution (OR = 1.1; 95% CI: 0.2–4.8).

## 4. Discussion

The proportion of IBV-positive samples detected by RT-PCR was 16.6% (15/90). The distribution of IBV under Ethiopian conditions has been understudied, emerging as a concern within the past few years. In 2017, IBV isolates belonging to a vaccinal genotype were identified through sequencing from a government-owned breeder farm in Bishoftu, likely the first report in the country [17]. Subsequent detection and genetic characterization were performed in 2022 in the southwest region, revealing isolates with a genetic composition similar to the first [18]. Recent studies in the Central Gondar Zone [24] and two districts of the East Shewa Zone [21] detected IBV using RT-PCR in swab samples.

Despite molecular detection, seropositive birds were found in the Ada'a District, Bishoftu, Bonga, Hawass, Kality, Holeta [17, 19–21, 25], and areas in Gondar and West Gojjam [24, 26]. These seroprevalence and viral detections across different geographical areas indicate the virus' expansion in a country with limited vaccination practices.

We found no significant difference in susceptibility to the virus between male and female birds. An experimental study comparing pathogenesis and host immune responses in one-week-old chicks showed no significant difference, except for minor disease severity in males [27]. Similarly, a Nigerian study reported nonsignificant differences in seroprevalence between sexes [28]. Therefore, despite physiological differences, both sexes appear equally susceptible to IBV.

It was found that susceptibility to the virus was not age-dependent, with similar virus prevalence in both age categories. A study experimentally infecting 3- and 10-week-old chicks revealed less prominent nephritogenic lesions in younger birds [29]. Another study reported longer viral clearance from various organs in older birds compared to younger birds, except for the trachea [30]. However, younger chickens have been reported to be more susceptible to oviduct-affecting strains [31]. These conflicting findings suggest that the most susceptible age might depend on viral tropism, pathogenesis (respiratory vs. nephropathic), and sample type (respiratory swab vs. kidney or oviduct tissue).

We observed no significant difference in virus distribution between exotic and local birds. In contrast, studies comparing inbred white Leghorn lines [32] and brown and white Leghorn lines [33] reported significant differences in susceptibility and mortality rates, respectively. Da Silva described the involvement of MHC alleles B2 and B18 in breed-related susceptibility to IBV [34]. Without evaluating these chromosomes and loci in our study breeds, resistance assessment can only be based on phenotypic performance.

Management risk factors such as carcass disposal, access to veterinary services, exposure to living fomites or carriers, and cleaning equipment practices could affect virus transmission. Birds whose owners disposed of carcasses in open fields were twice as likely to be infected (22.2%) compared to those where carcasses were fed to pets (11.1%). The importance of proper carcass disposal in preventing pathogen spread was highlighted by Blake during the 2002 Virginia outbreak of low pathogenic avian influenza, where infected carcass movement was implicated in virus dissemination [35, 36]. Open carcass disposal can disrupt disease control and create infection cycles.

Access to veterinary services, including deworming, antibiotic treatment, and micronutrient supplementation, indirectly impacts poultry health. Birds receiving treatment for various conditions were less likely to be infected compared to those without treatment. Deworming [37, 38], antibacterial treatment [39] and micronutrient supplementation [40] have been reported to enhance viral immunity against NDV and IBV. Therefore, the lower IBV prevalence among treated birds is unsurprising.

Chicken residing in premises accessible to wild birds and rodents exhibited higher susceptibility to IBV compared to those in isolated environments. The detection of IBV antibodies in free-living pigeons and Japanese quails in Nigeria and the detection of wild-type strains in Brazil indicate their potential as viral reservoirs [41, 42]. Rodents are well-known vectors for various pathogens [43], and their role in disease transmission could extend beyond currently recognized pathogens.

While enveloped viruses, including coronaviruses, are generally susceptible to detergents [44], the effectiveness of cleaning practices can vary based on the cleaning agent and frequency. Daily cleaning of feeders with diluted bleach solution has shown to reduce parasite burden in finches [45]. However, aerosol infection and environmental contamination might override the impact of cleaning feeders and drinkers in viral disease control.

## 5. Conclusion

Although limited studies have been conducted, the detection of IBV in various regions of Ethiopia, including the southwest, confirms the virus' widespread distribution. Serological findings further support the expansion of IBV within the country. These results emphasize the urgent need for enhanced biosecurity measures and comprehensive research to develop effective IBV control strategies in Ethiopia.

## Figures and Tables

**Table 1:** Association of risk factors with detection of the virus.

Factors		Positive (%)	Total (%)	Chi-square	*P* value	OR	CI (95%)	
							Upper	Lower
Sex	Female	7 (14.3)	49 (100)	0.43	0.57	0.68	0.22	2.09
	Male	8 (19.5)	41 (100)			1		

Breed	Exotic	8 (19)	42 (100)	0.321	0.586	1.37	0.4	4.1
	Local	7 (14.6)	48 (100)			1		

Age	Adult	7 (15.9)	44 (100)	0.036	1	0.9	0.29	2.72
	Young	8 (17.4)	46 (100)			1		

Site	Seqa Chekorsa	9 (20)	19 (100)			1.91	0.31	5.59
	Nadhigibe	3 (11.5)	26 (100)			1.33	0.46	7.83
	JUCAVM	3 (15.8)	45 (100)			1		

Fate of dead chicken	Left for pets	5 (11.1)	45 (100)	2	0.25	0.43	0.13	1.40
	Thrown to fields	10 (22.2)	45 (100)			1		

Access to veterinary services	Yes	6 (11.1)	36 (100)	3	0.09	2.7	0.8	8.3
	No	9 (25)	54 (100)			1		

Entrance of wild birds and rodents	Yes	3 (42.9)	7 (100)	3.74	0.088	4.4	0.88	22.3
	No	12 (14.5)	83 (100)			1		

Cleaning of feeder/drinker	No	3 (18.8)	16 (100)	0.061	0.726	1.19	0.29	4.83
	Yes	12 (16.2)	74 (100)			1		

## Data Availability

The data and materials used in this study are available upon reasonable request.

## Appendix

### A. Questionnaire for Assessment of Avian Infectious Bronchitis Virus Infection in Chicken during Sample Collection

Part I: General Information.

Date _______________

Sample Id: ---------------------------------

  (1) Region______________Zone_________________Town ______________
   Keble ___________
  (2) Name of the respondent: _________________Age: ________Sex: __________
  Level of education: ___________Number of chicken: ___________

Part II: Detail on flock management information.

  (3) Flock type and age of chicken (on the day of visit)

  (4) How far the poultry farm from neighboring one?
  1. <10M 2. 10–20M 3. 20−40M 4. >40M.
  (5) Is there any iterance of wild bird/rodents? (1. Yes 2. No)
  (6) Do you clean the chicken house/waste? (1. Yes 2. No)
  (7) Do you wash/clean drinking equipment? (1. Yes 2. No)
  (8) Do you use antiseptics/disinfectant before enter into house (1. Yes 2. No)
  (9) Where is the source of feed supplement? 1. Market 2. Home 3. Others
  (10) Where is the source of water for your chicken?
  (11) Top water 2. Bore water 3. Hand dug well 4. River 5. Pond 6. Others

Part III: About disease and vaccination

  (12) Do you ever see, chicken loss in your flock in the last three months?
      1. Yes   2. No
  (13) What type of chicken disease commonly observed in the farm?
   1. Respiratory sign 2. Nervous sign 3. Git sign 4.other
  (14) At which season you frequently faced disease in farm/area?
   1. Winter 2. Autumn 3. Summer 4. Spring
  (15) Which age group is affected? (1. <3 months 2. 3–5 month 3.>5 month)
  (16) What is done with sick chicken (possible to select many?)
   1. Isolated from health one 2. Eaten 3. Sold 4. Others
  (17) What is done with dead chicken?
   1. Buried 2. Burnt thrown away in to garbage 3. Eaten to dog or cat 4. Thrown away into ditch roadside 5. Others
  (18) Do you have access to veterinary service for your chicken? (1. Yes 2. No)
  (19) Did you vaccinate your flock against any chicken disease in last three months?
     (1. Yes 2. No).
  (20) If 18 is yes could you mentioned the disease, type and frequency of vaccination

  Administration form ^∗^ 1. drinking water 2. Intraocular 3. Injections.
  (21) Current status of chicken vaccination for IBV? (1. Yes 2. No)
  (22) What are the main problems of farm? 1. Disease 2. Water 3. Feed 4. House 5. All

Checklist of format used during sample collection.

## Abbreviations

RT-PCR: Reverse transcription polymerase chain reaction
IBV: Infectious bronchitis virus
OR: Odds ratio.
